# Salvage Frenuloplasty With Porcine Small Intestinal Submucosa Graft in a Newborn Following Unsuccessful Laser Release: A Case Report

**DOI:** 10.1155/crot/6192981

**Published:** 2026-04-19

**Authors:** Carlos O’Connor-Reina, Maria Teresa Garcia Iriarte, Laura Rodriguez Alcala, Eduardo Correa, Peter Baptista, Guillermo Plaza

**Affiliations:** ^1^ Otorhinolaryngology Department, Hospital Quironsalud Marbella, Marbella, 29680, Spain; ^2^ Otorhinolaryngology Department, Hospital Quironsalud Campo de Gibraltar, Palmones, Spain; ^3^ Otorhinolaryngology Department, Hospital Universitario Virgen de Valme, Sevilla, Spain; ^4^ Otorhinolaryngology Department, Clinica Universitaria de Navarra, Pamplona, Spain, cun.es; ^5^ Otorhinolaryngology Department, Hospital Universitario de Fuenlabrada, Universidad Rey Juan Carlos Madrid, Madrid, Spain; ^6^ Otorhinolaryngology Department, Hospital Sanitas la Zarzuela, Madrid, Spain

**Keywords:** ankyloglossia, breastfeeding, frenotomy, frenuloplasty, porcine small intestinal submucosa, postoperative scarring, scarring, tongue-tie

## Abstract

**Introduction:**

As the number of frenotomy procedures increases worldwide, the recognition and management of potential complications has become increasingly relevant. Postprocedural scarring following laser release may severely restrict tongue mobility in infants and occasionally requires surgical revision.

**Case Presentation:**

We report the case of a 3‐month‐old infant referred for persistent feeding difficulties and poor weight gain (5th percentile) after an unsuccessful laser frenotomy performed elsewhere. Clinical examination revealed severe ankyloglossia secondary to a dense restrictive scar, with a Bristol Tongue Assessment Tool (BTAT) score of 0. Revision surgery was performed under general anesthesia, consisting of complete excision of fibrotic tissue and reconstruction of the ventral tongue using a porcine small intestinal submucosa (SIS) graft to cover the mucosal defect. Intralesional corticosteroid therapy was not considered due to the absence of regulatory approval and limited safety data for intralesional use in infants with oral mucosal fibrosis. At 6‐week follow‐up, the infant demonstrated restored tongue mobility, normal breastfeeding, improvement of BTAT score to 6, and weight gain to the 50th percentile. At 12‐month follow‐up, the patient demonstrated sustained functional improvement, normal oral feeding without restriction, appropriate weight gain for age, and no recurrence of fibrotic scarring or tongue mobility limitation.

**Discussion:**

In this case, the extent of fibrosis precluded tension‐free primary closure. The use of a porcine SIS graft facilitated mucosal coverage and aimed to reduce the risk of recurrent scarring. Given the limited pharmacological options available for neonatal fibrosis management, selected surgical reconstruction strategies may represent a reasonable alternative in severe cases.

**Conclusion:**

Porcine SIS grafting may be considered in selected infants presenting with severe postfrenotomy scarring when conventional closure is not feasible. Longer follow‐up and additional cases are needed to establish safety and reproducibility.

## 1. Introduction

Ankyloglossia, or tongue‐tie, is a congenital oral anomaly characterized by a restrictive lingual frenulum that can interfere with breastfeeding, speech development, and orofacial growth. The worldwide diagnosis and treatment of this condition has increased substantially over the past two decades, especially among infants and young children [1]. Large‐scale population data suggest that pediatric ankyloglossia affects up to 8% of newborns, although the prevalence estimates vary widely due to inconsistent diagnostic criteria and differences in awareness among clinicians [1].

Despite its growing recognition, there is still no universally accepted definition or classification system for ankyloglossia, and the correlations between anatomical findings and functional impairment remain unclear. Several classification tools, such as the Kotlow and Hazelbaker classifications and the Bristol Tongue Assessment Tool (BTAT) [2], are used inconsistently in different settings, and fewer than half of clinicians regularly incorporate them into clinical decision‐making. Moreover, prognostic indicators to identify which patients may benefit most from surgical intervention are lacking, and long‐term outcome data remain scarce [2].

Management strategies also vary significantly between providers. A recent survey of pediatric otolaryngologists, dentists, and surgeons revealed substantial heterogeneity in diagnostic approaches, procedural techniques, sedation practices, and postoperative care. Although some clinicians favor sharp dissection under general anesthesia, others routinely use laser techniques without sedation in the outpatient setting. Similarly, recommendations for postprocedural exercises and referrals to adjunctive therapies, such as speech or myofunctional therapy, differ markedly between disciplines [3].

Frenotomy is described as a quick and low‐risk procedure for this condition, but recent studies suggest the need for a more cautious view, especially in newborns. For example, a 24‐month national surveillance study in New Zealand identified complications serious enough to require hospital admission in most cases involving frenotomy. The most common complications were poor feeding, apnea, bleeding, and weight loss. In some infants, the focus on the tongue‐tie delayed the diagnosis of underlying conditions such as cardiac defects or severe dehydration [4].

These complications have been described in heterogeneous clinical settings, often involving procedures performed by practitioners from different backgrounds and outside hospital environments. Reports of excessive scarring, feeding difficulties, and oral aversion following laser frenotomy are primarily derived from observational studies and healthcare professional surveys, which may be subject to reporting bias and limited sample sizes [5]. Therefore, while such complications appear to occur, the true incidence and causative factors remain incompletely defined.

In certain complex cases—particularly following prior laser frenotomy—dense scar tissue can significantly restrict tongue mobility and complicate revision surgery. When primary closure is not feasible, less conventional reconstructive strategies may be required. In this context, porcine small intestinal submucosa (SIS) grafts, which have been used successfully in other areas, such as otolaryngology, urology, and neurosurgery [6–8], offer a practical alternative for covering the surgical wound and helping to prevent readhesion of the frenulum. Although their use in newborns is not well documented, their biocompatibility and capacity for integration into host tissue make them a potential alternative in selected cases.

## 2. Case Presentation

We report the case of a 3‐month‐old infant who presented with recurrent ankyloglossia caused by dense fibrotic tissue formation following a previous laser frenotomy. This work has been reported in line with the SCARE criteria [9]. No artificial intelligence was used for data collection, patient care, or analysis.

**FIGURE 1:**
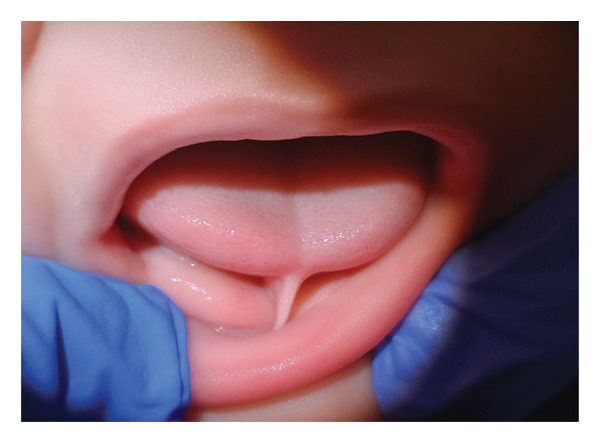
Preoperative view of the infant’s tongue showing a restrictive fibrotic lingual frenulum following a previous laser frenotomy. The limited elevation and anterior extension are consistent with a BTAT score of 0.

The infant was at the 5th percentile for body weight and exhibited marked difficulties in breastfeeding and bottle feeding, digestive discomfort (excessive gas), and fragmented sleep patterns, likely secondary to feeding inefficiency and frequent awakenings. The infant was born at term following an uncomplicated pregnancy and vaginal delivery. There was no relevant perinatal history, no congenital anomalies, and no previous medical or surgical conditions. No family history of feeding disorders was reported. Clinical examination revealed restricted tongue elevation and protrusion with a BTAT score of 0 [10], limiting effective latch during breastfeeding and resulting in prolonged feeding times, poor milk transfer, and maternal nipple discomfort. Bottle feeding was also inefficient, with frequent pauses and air swallowing, contributing to digestive discomfort. The mother reported adequate milk supply and no history of mastitis or nipple trauma. On palpation, a clinical dense fibrotic scarring component was identified in association with muscular ankyloglossia [11]. The mother reported a personal history of keloid scarring (Figure 1).

Given the severity of feeding and developmental concerns, a surgical revision under general anesthesia was promptly performed. General anesthesia was induced with inhalational sevoflurane and maintained with a balanced anesthetic technique. Airway management was achieved by orotracheal intubation to ensure optimal surgical exposure and airway protection, and the endotracheal tube was secured laterally to optimize exposure of the ventral tongue and the floor of the mouth. Standard pediatric monitoring was applied throughout the procedure, including continuous electrocardiography, pulse oximetry, capnography, and noninvasive blood pressure monitoring. Intraoperative analgesia was provided with intravenous fentanyl and paracetamol according to weight‐based dosing. The procedure was completed without anesthetic complications.

Intraoperatively, dense fibrotic tissue was released, exposing the genioglossus muscle using cold dissection and bipolar hemostasis.

Given the extensive scarring, the mucous layer could not be separated from the underlying muscle layers. To prevent tension on the suture, the wound was intentionally left open and covered with a porcine SIS graft to prevent readhesion and control bleeding.

**FIGURE 2:**
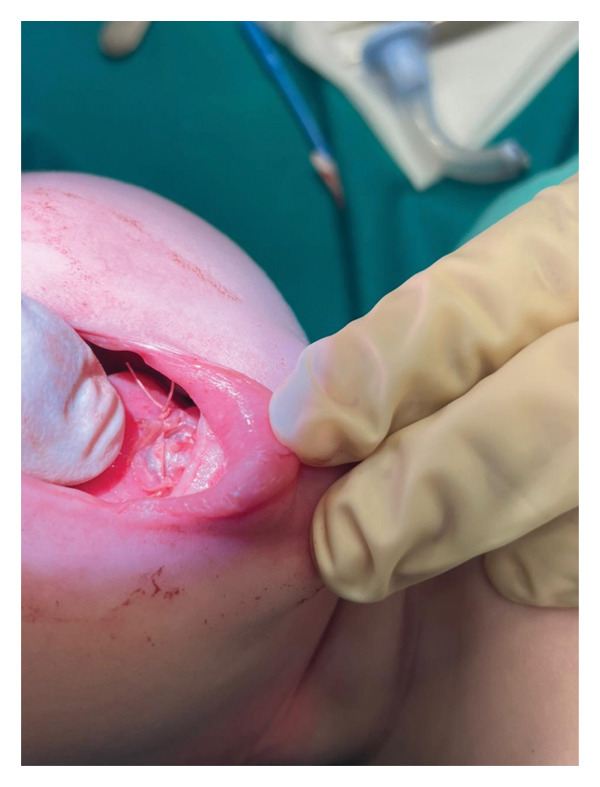
Immediate postoperative view showing the surgical field after complete excision of the dense fibrotic scarring. The porcine SIS graft is shown in place covering the exposed ventral tongue surface to prevent readhesion and promote second‐intention healing.

An immediate postoperative view of the SIS graft in place during closure is shown in Figure 2. Closure was completed using 4‐0 vicryl sutures under microscopic guidance. The infant awoke without complications, and the postoperative course was uneventful.

**FIGURE 3:**
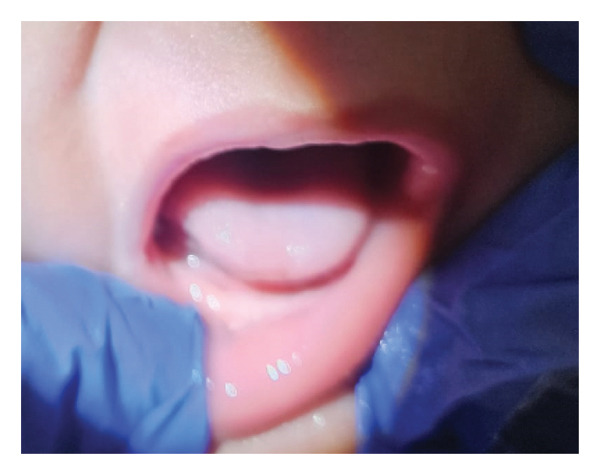
Postoperative view at the 6‐week follow‐up showing restored tongue mobility and the absence of fibrotic adhesions. The infant demonstrated normal breastfeeding, an improved BTAT score (from 0 to 6), and significant weight gain (from the 5th to 50th percentile).

By the 6‐week follow‐up, breastfeeding had normalized, body weight had increased to the 50th percentile, and the BTAT score had improved to 6 (Figure 3).

At 12‐month follow‐up, the patient demonstrated sustained functional improvement, normal oral feeding without restriction, appropriate weight gain for age, and no recurrence of fibrotic scarring or tongue mobility limitation.

## 3. Discussion

One of the main limitations of tongue‐tie surgery is the lack of appropriate technique selection based on the specific type of ankyloglossia. In most published articles, the selection of the technique has relied primarily on anatomical classification [12, 13] or used a single surgical approach regardless of the frenulum’s composition (i.e., mucosal, fibrotic, or muscular) [11]. In our experience, each type of ankyloglossia requires an individualized approach, independent of its anatomical appearance. Treatment decisions should be made based on the tissue characteristics and not solely on classification systems. Specifically, laser surgery may be less suitable in cases where muscular involvement is suspected because it may increase the risk of fibrotic formation and makes adequate dissection unfeasible in an office‐based procedure, particularly in newborns. Although histologic confirmation was not obtained, the rapid and excessive fibrotic response observed clinically resembled keloid‐like behavior.

Z‐plasty‐based frenuloplasty, with or without genioglossus myotomy, is a well‐established technique designed to lengthen the ventral tongue mucosa and reduce postoperative contracture. Recent studies have demonstrated improved functional outcomes and measurable increases in oropharyngeal airway dimensions following Z‐plasty closure compared with simple linear closure [14]. However, in the present case, the extensive fibrotic scarring and significant mucosal deficiency precluded the safe design of viable mucosal flaps. The ventral tongue mucosa could not be adequately mobilized from the underlying muscle without generating excessive tension, and flap creation in a scarred, poorly vascularized field would have increased the risk of necrosis or recurrent contracture. Given the patient’s age and limited tongue size, an open reconstruction with biological interposition was considered a more appropriate strategy to minimize tension and reduce the risk of readhesion.

The principle of interposing a biological barrier to prevent fibrosis and recurrence after frenuloplasty has been previously described by Godley in his “How I Do It” series, where a buccal mucosal graft was used to reconstruct the ventral tongue surface following frenulum release [15]. This approach demonstrated that grafted mucosa facilitates second‐intention healing while minimizing scar contracture. In our case, the same concept was applied using a porcine SIS graft, which provided a readily available and biocompatible alternative when autologous tissue harvesting was not feasible in an infant.

**FIGURE 4:**
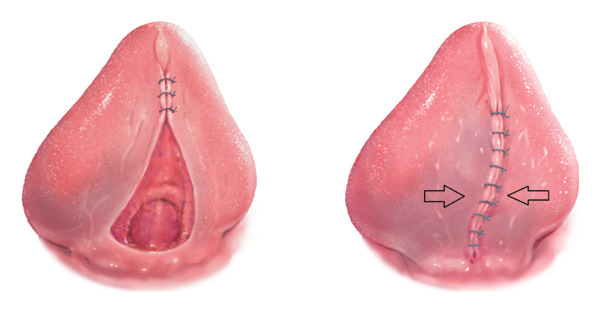
Conventional closure leaves the intact wound edges under opposing tension (right), which promotes fibrosis and recurrence.

One of the main concerns with the conventional surgical treatment of ankyloglossia is that the use of a scalpel removes the tension by cutting the fibrous structures but leaves both wound edges intact. This often triggers the body’s inflammatory response to attempt to repair the tissue and may lead to fibrosis and recurrence of restriction (Figure 4) [16].

In the present case, the use of a porcine SIS graft helped minimize tension between the wound edges (Figure 5), which promoted second‐intention healing and reduced the risk of readhesion and bleeding.

FIGURE 5(a) Placement of the porcine SIS graft over the open wound, extending approximately 5 mm beyond the wound margins. (b) Graft sutured in place without tension, as indicated by the arrows.

**Figure:**
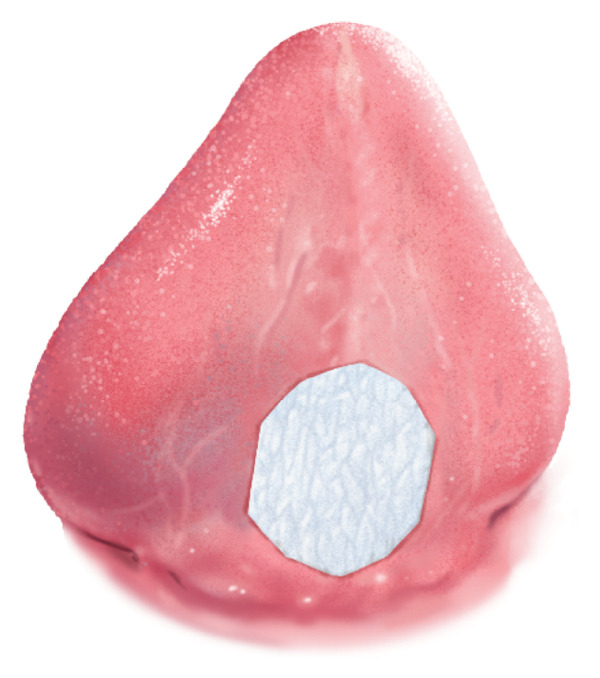
(a)

**Figure:**
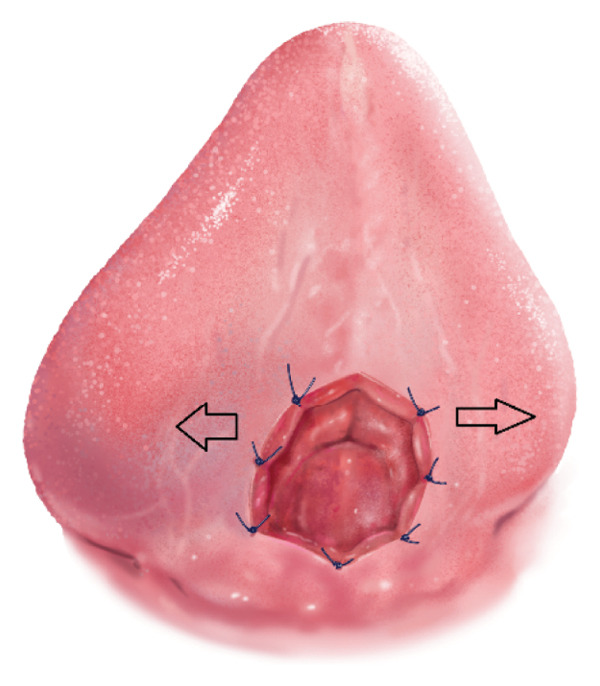
(b)

The Biodesign Repair Graft (Bloomington USA) is a porous biomaterial comprising a laminated extracellular collagen matrix derived from porcine SIS. This is obtained from the intestine using a process that retains the natural composition of matrix molecules such as collagen (Types I, III, and VI), glycosaminoglycans (hyaluronic acid, Chondroitin Sulfate A and B, heparin, and heparin sulfate), proteoglycans, and fibronectin.

Porcine SIS grafts are widely used in various surgical procedures. These grafts are effective in covering mucosal defects in different regions of the body, and no significant complications of porcine SIS grafts have been reported [17]. Such grafts are used to repair tympanic perforations in children [18]. To our knowledge, this is the first reported use of a porcine SIS graft to treat a failed frenuloplasty. In addition to xenogeneic collagen matrices, synthetic graft materials have been described in reconstructive oral procedures. However, their use in infants is limited, and concerns remain regarding biocompatibility, foreign body reaction, and integration within delicate oral mucosa. In the present case, a porcine collagen matrix was selected due to its established biocompatibility, resorbable nature, and favorable handling properties in pediatric oral surgery.

In cases of keloid formation, intralesional triamcinolone is considered to be a therapeutic option [19]. However, its use is not recommended in newborns [20], which leave few alternative treatments.

Given the increasing number of frenuloplasties being performed by a heterogeneous group of practitioners [21], it is likely that the need for rescue surgeries will continue to increase. Therefore, new therapeutic options should be explored. In our experience, the use of alternative materials such as porcine SIS grafts represents a reasonable approach to avoid potential complications.

The main limitations of this report are its basis on a single case and the cost associated with the graft material. Prospective randomized clinical trials are needed to confirm the effectiveness and reproducibility of this approach.

## 4. Conclusion

This case highlights that reconstruction using porcine SIS graft may represent a potential option in selected cases of severe scarring following failed frenotomy when conventional techniques are not feasible. However, longer follow‐up and additional cases are necessary to evaluate durability, safety, and reproducibility. Comparative studies would be required before drawing conclusions regarding superiority or broader generalizability of this approach.

## Funding

This research did not receive any specific grant from funding agencies in the public, commercial, or not‐for‐profit sectors.

## Ethics Statement

This study describes a single clinical case managed as part of routine medical care, using a commercially available and approved surgical material. Isolated case reports do not require formal review or approval according to the current regulations. Written informed consent was obtained from the patient’s legal guardian for both the surgical procedure and the publication of the case details and accompanying images. The porcine SIS graft used (Biodesign Repair Graft, Cook Medical, USA) is CE‐marked for surgical use in soft‐tissue repair. Although not specifically approved for infants, its use in this case was based on surgeon discretion and prior evidence of biocompatibility in pediatric applications.

## Consent

Written informed consent was obtained from the patient’s parents/legal guardian for publication and any accompanying images. A copy of the written consent is available for review by the Editor‐in‐Chief of this journal on request.

## Conflicts of Interest

The authors declare no conflicts of interest.

## Data Availability

The data that support the findings of this study are available on request from the corresponding author. The data are not publicly available due to privacy or ethical restrictions.
